# Cohort profile: DNA methylation in the Northern Ireland Cohort for the Longitudinal Study of Ageing (NICOLA) – recruitment and participant characteristics

**DOI:** 10.1136/bmjopen-2024-085652

**Published:** 2024-09-13

**Authors:** Claire Potter, Claire Hill, Laura J Smyth, Charlotte Neville, Angela Scott, Frank Kee, Bernadette McGuinness, Amy McKnight

**Affiliations:** 1 Centre for Public Health, Queen's University Belfast, Belfast, UK

**Keywords:** MOLECULAR BIOLOGY, EPIDEMIOLOGIC STUDIES, Aging, BIOTECHNOLOGY & BIOINFORMATICS, EPIDEMIOLOGY

## Abstract

**Abstract:**

**Purpose:**

Epigenetic modifications including DNA methylation (DNAm) are proposed mechanisms by which social or environmental exposures may influence health and behaviours as we age. The Northern Ireland Cohort for the Longitudinal Study of Ageing (NICOLA) DNAm cohort, established in 2013, is one of several worldwide, nationally representative prospective studies of ageing with biological samples from participants who consented to multiomic analysis.

**Participants:**

NICOLA recruited 8478 participants (8283 aged 50 years or older and 195 spouses or partners at the same address aged under 50 years). Computer-Assisted Personal Interviews, Self-Completion Questionnaires and detailed Health Assessments (HA) were completed. Of the 3471 (44.1%) participants who attended the HA in wave 1, which included venous blood sampling, 2000 were identified for the DNAm cohort. Following technical and data quality control checks, DNAm data are currently available for n=1870.

**Findings to date:**

There was no significant difference based on age, self-reported gender, education, employment, smoking or alcohol status and subjective health reports between the DNAm cohort and other HA attendees. Participants were more likely to be in the DNAm group if they lived with one other person (OR 1.26, 95% CI 1.07 to 1.49). The DNAm group had a lower proportion of depressed participants and those meeting criteria for post-traumatic stress disorder (11.7% and 4.4% vs 13.5% and 4.5%, respectively) categorised by objective assessment tools but this was not significant (OR 0.84, 95% CI 0.69 to 1.02 and OR 0.87, 95% CI 0.64 to 1.19).

**Future plans:**

The deeply phenotyped DNAm cohort in NICOLA with planned prospective follow-up and additional multiomic data releases will increase the cohort’s utility for research into ageing. The genomic and epigenetic data for the DNAm cohort has been deposited on the European Genome-Phenome Archive, increasing the profile of this cohort and data availability to researchers.

**STRENGTHS AND LIMITATIONS OF THIS STUDY**

- The Northern Ireland Cohort for the Longitudinal Study of Ageing (NICOLA), established in 2013, is one of several worldwide, nationally representative prospective studies that capture DNA methylation (DNAm) alongside detailed assessments of health and well-being.
- Participants were recruited to the DNAm cohort based on attendance at the NICOLA wave 1 health assessment and availability of high-quality DNA extracted from buffy coats of whole blood processed locally with consent included for other multiomic approaches.
- Omic data for this cohort has been deposited on the European Genome-Phenome Archive with a corresponding data description note under review. Phenotype information can be accessed following NICOLA Data Access Committee approval, see https://www.qub.ac.uk/sites/NICOLA/InformationforResearchers/.
- A limitation of this work for generalisability internationally is that all of the DNAm cohort are of white European ancestry, but this reflects the demographics of the Northern Ireland population.

## Introduction

 Population-based studies of older individuals have been established around the world to provide nationally representative, longitudinal studies of ageing to illuminate how we might age well. These include the ‘family’ of harmonised international ageing studies based on the Health and Retirement Study (HRS) established in North America in 1992^1^ designed to capture detailed objective and subjective measures of socioeconomic status, mental well-being and health outcomes longitudinally. The Northern Ireland Cohort for the Longitudinal Study of Ageing (NICOLA) launched as Northern Ireland’s (NI) largest health and social care cohort with approximately 8500 community-dwelling participants aged 50 years or older recruited to undergo wave 1 assessment between December 2013 and March 2016 with follow-up surveys planned every 2 years and detailed health assessment (HA) every 4 years.^2^ Participants complete a Computer-Assisted Personal Interview (CAPI), Self-Completion Questionnaire (SCQ) and are invited to attend a HA including blood sampling.^3^ Unique to NICOLA is the inclusion of detailed questions about the impact of a period of prolonged civilian conflict known as ‘The Troubles’ with objective measures of mental health such as the Centre for Epidemiologic Studies Depression scale (CES-D)^4^ and Post-Traumatic Stress Disorder checklist - civilian version (PCL-C).^5^

Investigation into the biological changes associated with environmental and social exposures across the life course within harmonised studies of ageing may help explain the variability seen in age-associated conditions.^6^ Epigenetics describes heritable and dynamic alterations in gene expression that are not caused by changes in the DNA sequence.^7^ Epigenetic modifications, such as DNA methylation (DNAm), are proposed as mechanisms by which social or environmental exposures experienced over the life course may influence behavioural traits and health outcomes.^8^ They can be used to study epigenome-wide associations between different phenotypic traits (known as epigenome-wide association studies (EWAS)) or as surrogates either for chronological age (termed biological age) or for health outcomes.^9^

A common challenge of nationally representative population-based cohort studies is potential selection bias or ‘healthy volunteer bias’, particularly for invasive HAs tasks or biobank sampling. In the UK Biobank, for example, a population-based cohort of 500 000 participants recruited in the UK between 2006 and 2010, participants were more likely to be older, female, not obese, be non-smokers and have fewer self-reported health conditions than non-participants.^10^ Even though there is an ongoing debate among epidemiologists about the relevance of representativeness for understanding causative mechanisms,^11^,^13^ sample weighting that is inversely proportional to participants probability of consenting to provide a blood sample as implemented in HRS with venous blood sample weight^14^ or careful study design permitting intentional selection based on specific demographic characteristics, as used in the Irish Longitudinal Study on Ageing (TILDA) DNAm cohort^15^ are some methods to overcome this issue. Detailed descriptions of cohort studies design, participant demographics and available biological samples will not only raise the profile of these valuable resources but provide researchers with the necessary information to determine if bias will influence their specific research question.^16 17^

## Cohort description

8283 community-dwelling adults, aged 50 years or older, were recruited from a randomised sample of NI addresses obtained from the Business Service Organization General Practitioner Register and stratified by geographical location and postcode, to generate a representative sample for NICOLA.^2^ Those who lacked the capacity to provide informed consent and or were institutionalised were not eligible to participate. If a spouse or partner also resided in the home at the time of the interview, regardless of aged over 50 they were also invited to participate (n=195) giving a final cohort size of 8478. Wave 1 CAPI interviews took place between December 2013 and July 2016 and included questions on employment, pensions, subjective health and care needs. 59.4% (n=5032) participants returned the SCQ which included questions on alcohol use, loneliness and previous traumatic events and 44.1% (n=3741) NICOLA participants attended the HA. Group characteristics between those who did and did not attend the HA have been previously reported^3^ but the majority of participants who attended the HA were aged 50–64 years, had reached secondary-level education, were married, retired and were a non-smoker.

**Figure 1:**
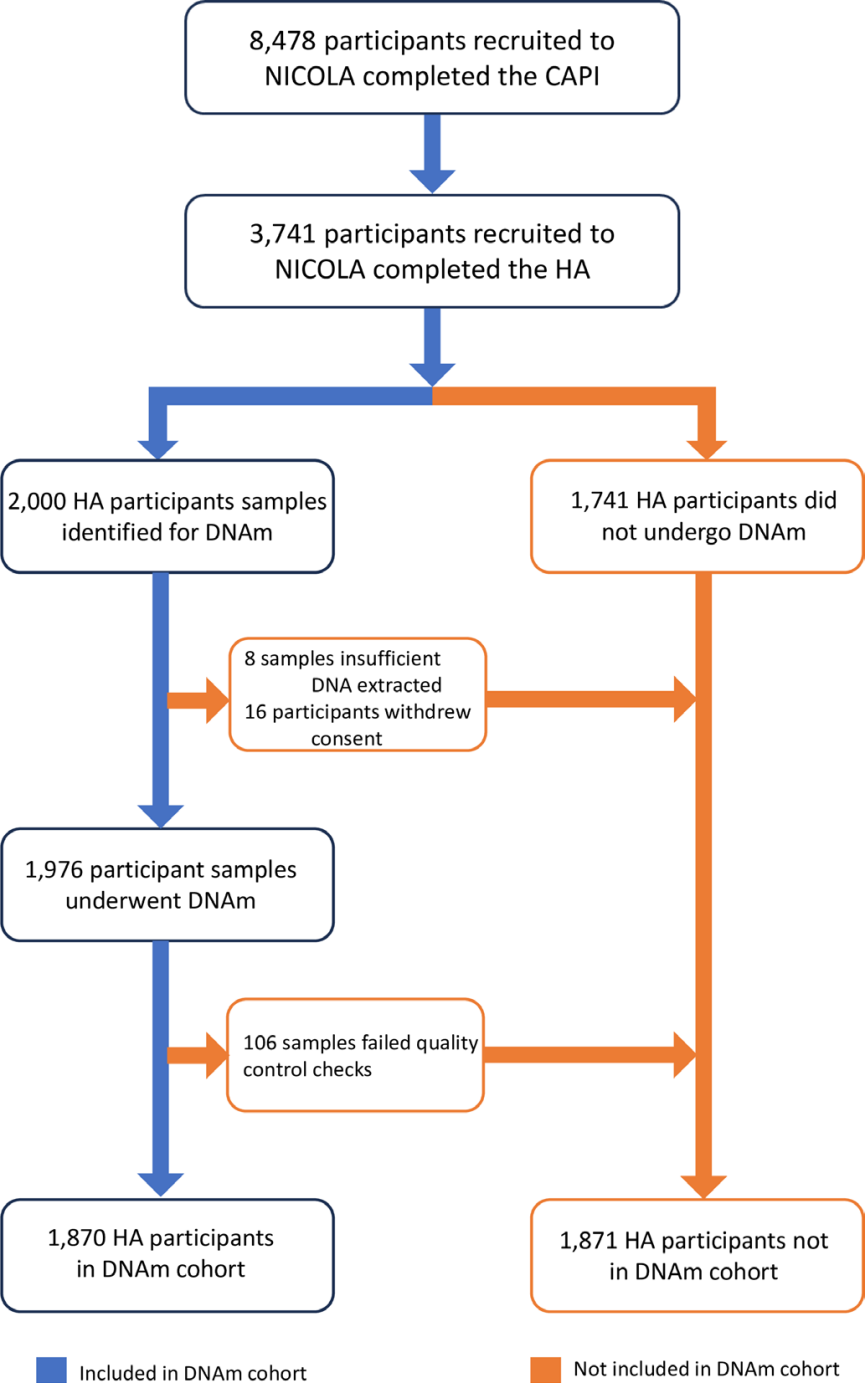
Flow chart of participant recruitment into DNAm cohort. CAPI, computer-assisted personal interview; DNAm, DNA methylation; HA, Health Assessment; NICOLA, Northern Ireland Cohort for the Longitudinal Study of Ageing.

Funding was available for 2000 participants to undergo DNAm. Samples were selected based on the first 2000 samples that had sufficient high-quality DNA extracted from buffy coats of whole blood of participants attending the HA^18^ processed in the Belfast City Hospital and is summarised in figure 1. To assess the methylation status of the CpG sites following bisulphite treatment, the Infinium MethylationEPIC BeadChip array (Illumina, USA) was used following the manufacturer’s instructions with samples randomly disrupted across each array. All samples were analysed together in the same laboratory and specifics of the laboratory methods and quality control (QC) processes have been described in detail.^18 19^ Eight samples were unsuitable as insufficient quantities of DNA were extracted and used as duplicates, 16 participants subsequently removed consent and requested their data be removed and 106 samples failed to pass the QC process giving a final DNAm sample size of 1870 (22.1% of NICOLA cohort).

Table 1 describes the selected baseline demographic, health behaviours and self-rated health for the DNAm group (n=1870) compared with the rest of the NICOLA participants that completed the CAPI at wave 1 (n=6608) described as the ‘no DNAm group’. Similar to findings in other longitudinal studies of ageing, differences in these group characteristics appear largely explained by the difference in group characteristics of those who do and do not attend HAs. Participants in the DNAm group were mainly 50–64 years age category, married, lived with at least one other person (majority their spouse or partner), were retired having achieved at least secondary-level education, were non-smokers and rated their subjective health, mental health and memory as good to excellent. The majority of participants when asked if they had been diagnosed with specific chronic health conditions, such as hypertension, cardiovascular disease, diabetes, arthritis or cancer reported none.

**Table 1:** Selected sociodemographic characteristics of NICOLA participants by DNAm group

	No DNAm groupn_max_=6608n (%)	DNAm groupn_max_=1870n (%)	P value
Age			
<50 years	154 (2.3)	40 (2.1)	<0.001
50–64 years	3214 (48.6)	953 (51.0)	
65–74 years	1885 (28.5)	621 (33.2)	
>75 years	1355 (20.5)	256 (13.7)	
Self-reported gender			
Male	2882 (43.6)	906 (48.4)	<0.001
Female	3726 (56.4)	964 (51.6)	
Household			
Lives alone	1813 (27.4)	352 (18.8)	<0.001
Lives with one person	3012 (45.6)	988 (52.8)	
More than one person	1783 (27.0)	530 (28.3)	
Marital status			
Married or living with partner	4206 (63.7)	1403 (75.0)	<0.001
Single	547 (8.3)	118 (6.3)	
Separated, divorced or widowed	1855 (28.1)	349 (18.7)	
Education			
Primary/none	1834 (27.8)	284 (15.2)	<0.001
Secondary	2934 (44.4)	800 (42.8)	
Higher	1765 (26.7)	785 (42.0)	
Employment			
Retired	3237 (49.0)	950 (50.8)	<0.001
Employed/self-employed (including farming)	2138 (32.4)	730 (39.0)	
Unemployed	205 (3.1)	38 (2.0)	
Permanently sick/disabled	553 (8.4)	66 (3.5)	
Looking after home/family/other*	393 (5.9)	81 (4.3)	
Deprivation score†			
0–0.11 (least deprived)	484 (7.3)	507 (27.1)	0.027
0.12–0.17	348 (5.3)	415 (22.2)	
0.18–0.23	377 (5.7)	356 (19.0)	
0.24–0.33	347 (5.3)	311 (16.6)	
>0.33 (most deprived)	271 (4.1)	239 (12.8)	
Smoking status			
Never	3088 (46.7)	987 (52.8)	<0.001
Current	2229 (33.7)	689 (36.8)	
Ex	1204 (18.2)	193 (10.3)	
Alcohol status			
Never	1383 (20.9)	312 (16.7)	<0.001
Current	3778 (57.2)	1293 (69.1)	
Ex	1357 (20.5)	264 (14.1)	
Subjective physical health			
Excellent	722 (10.9)	278 (14.9)	<0.001
Very good	1530 (23.2)	577 (30.9)	
Good	1858 (28.1)	537 (28.7)	
Fair	1552 (23.5)	340 (18.2)	
Poor	880 (13.3)	137 (7.3)	
Subjective mental health			
Excellent	1186 (17.9)	440 (23.5)	<0.001
Very good	1975 (29.9)	641 (34.3)	
Good	2108 (31.9)	520 (27.8)	
Fair	945 (14.3)	213 (11.4)	
Poor	324 (4.9)	55 (2.9)	
Subjective memory			
Excellent	641 (9.7)	181 (9.7)	0.006
Very good	1640 (24.8)	505 (27.0)	
Good	2417 (36.6)	706 (37.8)	
Fair	1373 (20.8)	388 (20.7)	
Poor	463 (7.0)	89 (4.8)	
Cardiovascular conditions‡			
None	3166 (47.9)	759 (40.6)	<0.001
One condition	2043 (30.9)	544 (29.1)	
2+conditions	1399 (21.2)	567 (30.3)	
Chronic health conditions§			
None	3224 (48.8)	969 (51.8)	<0.001
One condition	2247 (34.0)	675 (36.1)	
2+conditions	1137 (17.2)	226 (12.1)	

* Other includes those in education/training. These categories were combined due to low cell counts.

† Based on the Northern Ireland Multiple Deprivation measure 2018.^27^

‡ Cardiovascular conditions variable generated from count of participants endorsed diagnosis of hypertension, angina, heart attack, congestive heart failure, diabetes, stroke, mini stroke or transient ischaemic attack, high cholesterol, heart murmur, atrial fibrillation and or abnormal heart rhythm.

§ Chronic health conditions variable generated from count of participants endorsed diagnosis of chronic lung disease, asthma, arthritis, osteoporosis, cancer or malignant tumour, stomach ulcers, varicose ulcers and or cirrhosis.

DNAmDNA methylationNICOLANorthern Ireland Cohort for the Longitudinal Study of Ageing

Table 2 presents the difference in selected characteristics, anthropometric measures and health outcomes captured during the HA for the DNAm cohort (n=1870) and attended HA but not in DNA cohort (n=1871). The OR and CIs of the participant being selected for the DNAm cohort have also been reported.

**Table 2:** Selected characteristics, anthropometric measures and health outcomes captured during the health assessment for the DNAm cohort (n=1870) and attended health assessment but not in DNA cohort (n=1871)

	HA but no DNAm groupn_max_=1871	DNAm groupn_max_=1870	P value	OR (CI_lower_, CI_upper_)
Age				
<50 years	34 (1.8)	40 (2.1)	0.689	ref
50–64 years	278 (14.9)	256 (13.7)		1.04 (0.67, 1.61)
65–74 years	946 (50.6)	953 (51.0)		1.14 (0.73, 1.77)
>75 years	613 (32.8)	621 (33.2)		1.09 (0.68, 1.73)
Self-reported gender				
Male	876 (46.8)	906 (48.4)	0.335	ref
Female	998 (53.3)	964 (51.6)		0.94 (0.82, 1.07)
Household				
Lives alone	408 (21.8)	352 (18.8)	0.017	ref
Lives with one person	907 (48.5)	988 (52.8)		1.26 (1.07, 1.49)
More than one person	556 (29.7)	530 (28.3)		1.1 (0.92, 1.33)
Marital status				
Married or living with partner	1326 (70.9)	1403 (75.0)	0.012	ref
Single	126 (6.7)	118 (6.3)		0.89 (0.68, 1.15)
Separated, divorced or widowed	419 (22.4)	349 (18.7)		0.79 (0.67, 0.92)
Education				
Primary/none	295 (15.8)	284 (15.2)	0.150	ref
Secondary	847 (45.3)	800 (42.8)		0.98 (0.81, 1.19)
Higher	727 (38.9)	785 (42)		1.12 (0.93, 1.36)
Employment				
Retired	909 (48.6)	950 (50.8)	0.180	ref
Employed/self-employed (including farming)	762 (40.7)	730 (39.0)		0.92 (0.80, 1.05)
Unemployed	50 (2.7)	38 (2.0)		0.73 (0.47, 1.12)
Permanently sick/disabled	82 (4.4)	66 (3.5)		0.77 (0.55, 1.08)
Looking after home/family/other*	67 (3.6)	81 (4.3)		1.16 (0.83, 1.62)
Deprivation score†				
0–0.11 (least deprived)	484 (25.9)	507 (27.1)	0.027	ref
0.12–0.17	348 (18.6)	415 (22.2)		1.14 (0.94, 1.38)
0.18–0.23	377 (20.1)	356 (19.0)		0.90 (0.74, 1.09)
0.24–0.33	347 (18.5)	311 (16.6)		0.86 (0.70, 1.04)
>0.33 (most deprived)	271 (14.5)	239 (12.8)		0.84 (0.68, 1.04)
Smoking status				
Never	968 (51.7)	987 (52.8)	0.765	ref
Current	697 (37.3)	689 (36.8)		0.97 (0.84, 1.11)
Ex	204 (10.9)	193 (10.3)		0.93 (0.75, 1.15)
Alcohol status				
Never	322 (17.2)	312 (16.7)	0.266	ref
Current	1252 (66.9)	1293 (69.1)		1.07 (0.90, 1.27)
Ex	296 (15.8)	264 (14.1)		0.92 (0.73, 1.16)
Subjective health				
Excellent	271 (14.5)	278 (14.9)	0.006	ref
Very good	479 (25.6)	577 (30.9)		1.17 (0.95, 1.44)
Good	584 (31.2)	537 (28.7)		0.90 (0.73, 1.10)
Fair	381 (20.4)	340 (18.2)		0.87 (0.70, 1.09)
Poor	154 (8.2)	137 (7.3)		0.87 (0.65, 1.15)
Subjective mental health				
Excellent	411 (22.0)	440 (23.5)	0.052	ref
Very good	583 (31.2)	641 (34.3)		1.03 (0.86, 1.22)
Good	598 (32.0)	520 (27.8)		0.81 (0.68, 0.97)
Fair	223 (11.9)	213 (11.4)		0.89 (0.71, 1.12)
Poor	55 (2.9)	55 (2.9)		0.93 (0.63, 1.39)
Subjective memory				
Excellent	191 (10.2)	181 (9.7)	0.404	ref
Very good	487 (26.0)	505 (27.0)		1.09 (0.86, 1.39)
Good	705 (37.7)	706 (37.8)		1.06 (0.84, 1.33)
Fair	372 (19.9)	388 (20.7)		1.10 (0.86, 1.41)
Poor	114 (6.1)	89 (4.8)		0.82 (0.58, 1.16)
Cardiovascular conditions‡				
None	911 (48.7)	759 (40.6)	<0.001	ref
One condition	622 (33.2)	544 (29.1)		1.05 (0.90, 1.22)
2+conditions	338 (18.1)	567 (30.3)		2.01 (1.71, 2.38)
Chronic health conditions§				
None	946 (50.6)	969 (51.8)	<0.0001	ref
One condition	621 (33.2)	675 (36.1)		1.06 (0.92, 1.22)
2+conditions	304 (16.2)	226 (12.1)		0.73 (0.60, 0.88)
CES-D¶				
Not depressed	1515 (81.0)	1558 (83.3)	0.091	ref
Depressed	253 (13.5)	219 (11.7)		0.84 (0.69, 1.02)
PCL_C PTSD DSM diagnosis**				
No	1198 (64.0)	1329 (71.1)	0.427	ref
Yes	85 (4.5)	82 (4.4)		0.87 (0.64, 1.19)
Troubles impact				
None	219 (11.7)	217 (11.6)	0.439	ref
A little bit	562 (30.0)	649 (34.7)		1.17 (0.94, 1.45)
Moderate amount	362 (19.3)	416 (22.2)		1.16 (0.92, 1.47)
Quite a bit	253 (13.5)	263 (14.1)		1.05 (0.81, 1.35)
Extreme amount	52 (2.8)	70 (3.7)		1.36 (0.91, 2.04)
MMSE score	28.4 (1.9)	28.6 (1.7)	<0.001	
MOCA score	25.1 (3.4)	25.7 (3.1)	<0.001	
Animal recall	18.6 (5.3)	19.5 (5.7)	<0.001	
Colour trails (seconds)	121.8 (44.4)	114.6 (38.6)	<0.001	
Grip strength (kg)	32.2 (12.0)	33.5 (11.8)	0.002	
Timed up and go (seconds)	10.1 (2.9)	10.0 (2.7)	0.154	
BMI (kg/m^2^)	29.2 (5.5)	28.7 (5.0)	0.001	

Values are unweighted mean (SD) or median (IQR) for continuous variables or n (%) for categorical variables.

* Other includes those in education/training. These categories were combined due to low cell counts.

† Based on the Northern Ireland Multiple Deprivation measure 2018.^27^

‡ Cardiovascular conditions variable generated from count of participants endorsed diagnosis of hypertension, angina, heart attack, congestive heart failure, diabetes, stroke, mini stroke or transient ischaemic attack, high cholesterol, heart murmur, atrial fibrillation and or abnormal heart rhythm.

§ Chronic health conditions variable generated from count of participants endorsed diagnosis of chronic lung disease, asthma, arthritis, osteoporosis, cancer or malignant tumour, stomach ulcers, varicose ulcers and or cirrhosis.

¶ Classification based on Centre for Epidemiological Studies Depression (CES-D) score 0–15 not depressed and score >16 at risk of depression.

** Classification based on Post-Traumatic Stress Disorder checklist civilian version (PCL-C) scoring and symptomatic thresholds of Diagnostic and Statistical Manual of Mental Disorders (DSM-4) clusters of reexperiencing, avoidance and heightened arousal.

BMIbody mass indexMMSEMini-Mental State ExaminationMOCAMontreal Cognitive Assessment

There were roughly equal proportions of participants based on age category and self-reported gender across both groups (83.4% aged over 65 years in the no DNAm group vs 84.2% in the DNAm group and 53.3% vs 51.6% female participants in respective groups). There was no significant difference between the groups based on education, employment, smoking or alcohol status, subjective health reports or impact of the NI Troubles. Participants were less likely to be included in the DNAm group if they were separated, divorced or widowed compared with married (OR 0.79, 95% CI 0.67 to 0.92) and more likely to be in the DNAm group if they lived with one other person (OR 1.26, 95% CI 1.07 to 1.49).

Based on the participants’ responses to questions asked in the CES-D, they were assigned to categories not depressed (score 0–15) and depressed or at risk of depression (score >16). Based on PCL-C response and Diagnosistic and Statistical Manual of Mental Disorders (DSM) 4 cluster criteria,^5^ PTSD was assigned if participants endorsed symptomatic response (scoring 3–5 on Likert scale) in at least 1 ‘B item’ (Q1–5) for reexperiencing, at least 3 ‘C items’ (Q6–12) for avoidance and at least 2 ‘D items’ (Q13–17) for arousal. The DNAm group had a lower proportion of depressed participants and those meeting DSM cluster criteria for PTSD (11.7% and 4.4% vs no DNAm group with 13.5% and 4.5%, respectively) but this was not significant (OR 0.84, 95% CI 0.69 to 1.02 vs OR 0.87, 95% CI 0.64 to 1.19).

## Findings to date

To date, the NICOLA DNAm cohort has contributed to an international EWAS examining diabetic kidney disease,^20^ participants risk^19^ and time preference,^21^ socioeconomic position^22^ and validation of a blood DNAm biomarker for predicting short-term risk of cardiovascular events.^9^

## Collaboration

At the time of submission, the genomic and epigenetic data for the DNAm cohort has been deposited on the European Genome-Phenome Archive (https://ega-archive.org/). NICOLA phenotype data are stored, maintained and accessed via a safe setting within the Centre for Public Health, Queen’s University Belfast. Researchers can apply for access to the data and biosamples by submitting a research proposal to the NICOLA Data Access Committee. For more information, including meta-data documentation, please refer to https://www.qub.ac.uk/sites/NICOLA/InformationforResearchers/. Core variables are archived on an ongoing basis with the UK Data Service,^23^ the UK Longitudinal Linkage Collaboration,^24^ the Dementias Platform UK^25^ and the Gateway to Global Ageing Data (G2G),^26^ to collaborate with the data-sharing and harmonisation initiatives facilitating cross-country research.

## Patient and public involvement

A ‘Healthy Ageing Research Advisory Group’ has been established within NICOLA to engage with older people, explore their experiences, insights and opinions and through this allow them to provide input into the direction of NICOLA. The group has contributed to the design and final approval of study materials including components of the HA and molecular analysis. While aware of the research plans to complete DNAm, this PPI group was not separately consulted at this time on this specific analysis.

## Strengths and limitations

NICOLA’s study design, namely its relatively large sample size, designed to capture nationally representative participants with prospective rich data resource and biorepository including a range of multiomic samples enables investigation and monitoring of ageing across NI with great potential of cross-country harmonisation with other international ageing cohort studies. Although ethnic minorities are included within the cohort, and reflective of the NI population estimates, the small number included within the NICOLA sample would not be enough to report statistically robust estimates. All genomic data within NICOLA is presented for white individuals of European ancestry.

## Data Availability

Data are available on reasonable request.
