# Supplementary material for: Cohort profile: DNA methylation in the Northern Ireland Cohort for the Longitudinal Study of Ageing (NICOLA) – recruitment and participant characteristics
Source: BMJ Open. 2024 Sep 13;14(9):e085652. doi: 10.1136/bmjopen-2024-085652 (PMC11404182; doi:10.1136/bmjopen-2024-085652)
Supplement: Supplementary file 1 [file bmjopen-2024-085652.reviewer_comments.pdf]

PEER REVIEW HISTORY

BMJ Open publishes all reviews undertaken for accepted manuscripts. Reviewers are asked to complete a checklist review form and are provided with free text boxes to elaborate on their assessment. These free text comments are reproduced below.

ARTICLE DETAILS

**TITLE (PROVISIONAL)**	Cohort Profile: DNA methylation in the Northern Ireland Cohort for the Longitudinal Study of Ageing (NICOLA): recruitment and participant characteristics
**AUTHORS**	Potter, Claire; Hill, Claire; Smyth, Laura J; Neville, Charlotte; Scott, Angela; Kee, Frank; McGuinness, Bernadette; McKnight, Amy

VERSION 1 - REVIEW

**REVIEWER NAME**	Marioni, Riccardo
**REVIEWER AFFILIATION**	University of Edinburgh Western General Hospital
**REVIEWER CONFLICT OF INTEREST**	NA
**DATE REVIEW RETURNED**	09-May-2024

**GENERAL COMMENTS**	This is a clear manuscript that describes the selection process for the DNA methylation dataset of the NICOLA cohort. I have no major comments but have listed a few (very) minor points below.<br><br>Abstract: “biorepository of participants” seems an odd phrase. I think of a biorepository as a lab/centre where biosamples are stored. I’d try to re-word this.<br><br>Abstract: “between [the] DNAm cohort and other health assessment attendees”. Adding “the” makes this easier to read, in my opinion.<br><br>Abstract: was this self-reported depression? Same goes for PTSD – was that using information from a questionnaire?<br><br>Abstract: “cohort’s utility to ageing researchers” I’d probably swap this for “cohort’s utility for research into ageing”<br><br>Intro: I don’t think you need commas after “around the world” or “the long-standing”. In general, I’d have a careful proof read for adding/removing commas.<br><br>Intro: Why give a single example of the HRS as a study of ageing? There are so many to choose from! I’d maybe cite an overview article instead. A recent example is: https://www.nature.com/articles/s41591-023-02784-9<br><br>Intro: Please spell out EWAS more clearly upon first use.<br><br>Intro: please can you expand on what is meant by venous blood sample weight. Is this an inverse probability weight for those that donated blood?<br><br>Methods: Why were the 8 samples unsuitable?

	Methods: I'd probably say "failed to pass quality control (QC) checks" instead of "were lost during…"<br><br>Methods: "Further details on the QC process will be published with a data description note currently under review and is available to be shared." The end of this sentence is a bit awkward to read.<br><br>Figure 1 hasn't presented very nicely in the pdf e.g., text is outside of the boxes or overwritten and some of the text is also very small to read. I would also add a label to describe the different coloured arrows.<br><br>Table 1: It would be nice to report the p-values to the same number of significant figures. Personally, I'd opt for 2 significant figures e.g., P = 0.XX for anything between 0.1 and 1; 0.0XX for anything between 0.1 and 0.01; and P = X.X x 10-X for anything smaller than this. The same goes for Table 2.<br><br>Results: Although the analyses are primarily descriptive in nature, it would be good to make the code publicly available on e.g., GitHub for reproducibility.

**REVIEWER NAME**	Verschoor, Chris P.
**REVIEWER AFFILIATION**	Health Sciences North
**REVIEWER CONFLICT OF INTEREST**	None
**DATE REVIEW RETURNED**	21-Jun-2024

**GENERAL COMMENTS**	The authors present a nice summary of the NICOLA cohort, specifically the subset of participants for whom DNA methylation data was derived. The manuscript is well-written and very straightforward, providing basic demographic characteristics of NICOLA, how the DNAm cohort compares to other subsets within NICOLA and a basic description of the methods. Although manuscripts of this nature are valuable, I found this example to be lacking information-wise. I was disappointed that detailed information about the laboratory procedures (eg. DNA extraction and assessment, facility where the DNA methylation arrays were run) and QC pipeline were not included and instead indicated as "published elsewhere". I thought there was also an opportunity here to at least show the distribution of some of the more popular epigenetic clock measures in the sub-sample and perhaps how they correlated with age. It is almost certain that this data will be published elsewhere, but it is nonetheless foundational and expected to be repeated. Without this type of additional information, the manuscript feels a little "thin". Below are additional comments that I hope are helpful. Thank you.<br><br>Figure 1: This figure needs to be revisited as text is spilling over in places and bulleted text is too small to read.<br>Table 1: Although it is important to understand how the DNAm group differs from the rest of the cohort, I think Table 1 would be better suited as supplementary data. The most important comparison in my mind is the DNAm vs. non-DNAm health assessment groups.<br>Table 2: Could the p-values be denoted as asterisks, or at least in non-scientific format with bolding for significant points. Also, the OR would be better presented as: OR (lower, upper CI).

	- it would be really useful to include a statement regarding the availability of data to researchers outside of the UK and even EU.

VERSION 1 – AUTHOR RESPONSE

Reviewer 1's Comments

1. Abstract: “biorepository of participants” seems an odd phrase. I think of a biorepository as a lab/centre where biosamples are stored. I’d try to re-word this.
Authors response: This sentence has been rephrased to "... with biological samples from participants who consented for multi-omic analysis."
2. Abstract: “between [the] DNAm cohort and other health assessment attendees”. Adding “the” makes this easier to read, in my opinion.
Authors response: Edit made.
3. Abstract: was this self-reported depression? Same goes for PTSD – was that using information from a questionnaire?
Authors response: Further detail provided to clarify that depression and post-traumatic stress disorder (PTSD) were classified based on objective assessment tools. Abstract word count precluded describing these in full, but they are described in detail in the main body of the manuscript in the "Cohort Description" section.
4. Abstract: “cohort’s utility to ageing researchers” I’d probably swap this for “cohort’s utility for research into ageing”
Authors response: Edit made.
5. Intro: I don’t think you need commas after “around the world” or “the long-standing”. In general, I’d have a careful proof read for adding/removing commas.
Authors response: Edits made and proofread completed.
6. Intro: Why give a single example of the HRS as a study of ageing? There are so many to choose from! I’d maybe cite an overview article instead. A recent example is: https://www.nature.com/articles/s41591-023-02784-9
Authors response: We wanted to emphasize to readers that the NICOLA study is part of the Health and Retirements "family" of international harmonised studies of ageing. Sentence has been edited to make this clearer.
The article suggested by the reviewer provides a current and comprehensive review of the use and validation of biomarkers in ageing across multiple cohort studies whilst also presenting associated challenges and recommendations. We believe it will be valuable to readers and so have included it in this section.
7. Intro: Please spell out EWAS more clearly upon first use.
Authors response: Edit made.
8. Intro: please can you expand on what is meant by venous blood sample weight. Is this an inverse probability weight for those that donated blood?
Authors response: Edit made to clarify that yes, the HRS venous blood sample weight was the inverse probability weight of participants consenting to blood donation.
9. Methods: Why were the 8 samples unsuitable?
Authors response: Edit made that there was insufficient DNA extracted for these 8 samples and so not included.
10. Methods: I’d probably say “failed to pass quality control (QC) checks” instead of “were lost during…”
Authors response: Edit made
11. Methods: “Further details on the QC process will be published with a data description note currently under review and is available to be shared.” The end of this sentence is a bit awkward to read.
Authors response: Edit made. Removed this last sentence as edits made earlier in paragraph.
12. Figure 1 hasn’t presented very nicely in the pdf e.g., text is outside of the boxes or overwritten and

some of the text is also very small to read. I would also add a label to describe the different coloured arrows.
Authors response: Figure 1 has been replaced.
13. Table 1: It would be nice to report the p-values to the same number of significant figures. Personally, I'd opt for 2 significant figures e.g., P = 0.XX for anything between 0.1 and 1; 0.0XX for anything between 0.1 and 0.01; and P = X.X x 10-X for anything smaller than this. The same goes for Table 2.
Authors response: P values in Table 1 and Table 2 have been edited to 3 decimal places and <0.001 was used for values less than that.
14. Results: Although the analyses are primarily descriptive in nature, it would be good to make the code publicly available on e.g., GitHub for reproducibility.
Authors response: Code used for this analysis has been returned to the NICOLA data manager and is available on request. Sentence edited to reflect this.

Reviewer 2's Comments
1. I was disappointed that detailed information about the laboratory procedures (eg. DNA extraction and assessment, facility where the DNA methylation arrays were run) and QC pipeline were not included and instead indicated as "published elsewhere".
Authors response: The primary focus of this submission is as a cohort profile. Some additional details on laboratory procedures have been included, but we note that this cohort paper complements a data note where lab procedures and methods are described in depth.
2. I thought there was also an opportunity here to at least show the distribution of some of the more popular epigenetic clock measures in the sub-sample and perhaps how they correlated with age. It is almost certain that this data will be published elsewhere, but it is nonetheless foundational and expected to be repeated. Without this type of additional information, the manuscript feels a little "thin".
Authors response: The focus of this paper is to characterise NICOLA's DNAm cohort, providing baseline information at recruitment for a cohort that will be followed up longitudinally, rather than presenting traditional research results. This important information advises prospective researchers the rationale for the cohort creation, how participants were recruited, how representative they were of the wider population, how data was collected and plans for future Waves of data collection.

3. Figure 1: This figure needs to be revisited as text is spilling over in places and bulleted text is too small to read.
Authors response: Figure 1 has been replaced.
4. Table 1: Although it is important to understand how the DNAm group differs from the rest of the cohort, I think Table 1 would be better suited as supplementary data. The most important comparison in my mind is the DNAm vs. non-DNAm health assessment groups.
Authors response: We have kept Table 1 within the main body text. Justification for this is that it shows that the DNAm cohort doesn't differ significantly from rest of NICOLA cohort beyond the characteristics of those that did and did not attend for health assessment. This will be valuable to readers who may wish to analyse the DNAm cohort.
5. Table 2: Could the p-values be denoted as asterisks, or at least in non-scientific format with bolding for significant points. Also, the OR would be better presented as: OR (lower, upper CI).
Authors response: P values in Table 1 and Table 2 have been edited to 3 decimal places and <0.001 was used for values less than that. In Table 2, odds ratios are now presented as OR (lower, upper CI).
6. It would be really useful to include a statement regarding the availability of data to researchers outside of the UK and even EU.
Authors response: The data sharing statement confirms that we are keen to maximise the utility of this resource. Data is available to researchers worldwide through NICOLA's established data and sample access committees. The link to request access is included directly in the manuscript.

We look forward to your further consideration of this submission. If you require any further information please contact.
